# Comparative evaluation on the masking ability of different tooth colored restoration over blackish discoloration caused by 38% silver diamine fluoride: an in -vitro study

**DOI:** 10.1038/s41405-025-00318-8

**Published:** 2025-03-17

**Authors:** Prajval Mahajan, Anupama Nayak P, Srikant Natarajan, Karuna Yarmunje Mahabala, Kiran N Baliga, Ashwin Rao, Charisma Thimmaiah

**Affiliations:** 1 Department of Pediatric and Preventive Dentistry, Manipal College of Dental Sciences, Mangalore, Manipal Academy of Higher Education, Karnataka Manipal, 576104 India; 2 Department of Oral Pathology and Microbiology, Manipal College of Dental Sciences, Mangalore, Manipal Academy of Higher Education, Karnataka Manipal, 576104 India; 3 Department of Pediatrics, Kasturba Medical College, Mangalore, Manipal Academy of Higher Education, Karnataka Manipal, 576104 India

**Keywords:** Minimal intervention dentistry, Paediatric dentistry

## Abstract

**Background:**

The application of Silver diamine fluoride is an effective approach in controlling dental caries. But the black discoloration caused by its application is unaesthetic and is of greater concern to the parents.

**Aim:**

To assess the potential color masking ability of tooth colored restorative material over discoloration caused by 38% SDF using a UV‒VIS-NIR spectrophotometer.

**Material and methods:**

20 extracted teeth were taken and randomly divided into 4 groups: Group A: 38% SDF + GIC; Group B: 38% SDF + RMGIC; Group C: 38% SDF+ Composite; Group D: 38% SDF+ Cention N. Two readings were recorded, one on application of 38% SDF and second after application of test materials using a UV‒VIS-NIR spectrophotometer.

**Result:**

The intragroup comparison for color masking ability (ΔE) for all four restorative materials revealed no statistically significant differences, with a test value of 1.168 and a p value of 0.353, and the highest mean was observed in Group A, i.e., 38% SDF + GIC (9.171966) **S**ignificant differences in color were observed, with more shifts toward yellow in group A (2.488 ± 2.957) and group D (1.686 ± 0.559) and more shifts toward green in groups B (−0.088 ± 0.34) and C (−0.062 ± 0.5). The mean lightness was greatest for Group C, i.e., the composite group (86.396 ± 3.741), and least for Group A, i.e., the GIC group (76.664 ± 8.213).

**Conclusion:**

All 4 restorative materials were equally effective in terms of color masking over 38% SDF discoloration.

## Introduction

Early childhood caries (ECC) is a burden on children, families, and society and requires multiple treatments and appointments. Additionally, treating these children is a great challenge. This invoked interest among pediatric dentists in the use of alternative methods for controlling dental caries; thus, silver diamine fluoride (SDF) has gained popularity [1]. SDF is an alkaline, colorless solution that combines fluoride and silver to form a combination with ammonia. SDF is more than just ordinary silver, ammonium, and fluoride ion salt. Instead, it is a coordination complex of mixed heavy metal halides. For a whil,e ammonia can maintain the solution’s concentration at that level. Because of their antibacterial qualities, silver compounds have a long history of use in both medicine and dentistry. There are several ways to utilize fluoride to stop and prevent dental cavities. Therefore, it has been hypothesized that the combined effects of silver and fluoride could both stop the progression of caries and inhibit the development of new caries at the same time [2].

SDF was approved and considered safe for use by the Food and Drug Administration in 2014 [3]. SDF has several advantages, such as being cost effective, being less invasive, having antibacterial effects of silver, and having anti-cariogenic effects of fluoride [4], and is also an effective approach for controlling dental caries in very young children, adolescents, and children with special health care needs. By attaching themselves to bacterial cell components and altering enzymes involved in both sugar uptake and carbohydrate metabolism, high concentrations of fluorides prevent the formation of biofilms. Inhibiting enzymatic activity, which affects metabolic processes, preventing the reproduction of bacterial DNA, and penetrating and destroying bacterial cell wall structures are the three ways that silver ions have an antibacterial effect. It has been demonstrated that SDF remineralizes dentine caries. The creation of calcium fluoride and silver phosphate, which help to raise pH and create fluoride reservoirs, is one suggested chemical response between SDF and tooth hydroxyapatite. The development of insoluble fluorapatite is facilitated by the subsequent dissolution of calcium and fluoride. The creation of nanoscopic metallic silver particles affixed to hydroxyapatite crystals has been shown to be another consequence of the reaction between SDF and hydroxyapatite [5].

However, the major disadvantage of SDF is the black discolouration of the arrested carious lesion. Therefore, parents should be informed beforehand regarding the staining [6], and most of the parents/caregivers are apprehensive about this and show their concern, especially with its application in the anterior teeth.

Permanent restorations are indicated after caries have arrested and are very important for the long-term success and retention of primary teeth. Glass ionomer cement (GIC), resin-modified GIC (RMGIC), composites, and Cention are commonly used restorative materials for primary teeth [7]. Placement of these restorations may mask this unfavorable discoloration caused by SDF, but which of these restorative materials is better or more effective in masking black discolouration without affecting the arrested caries is unknown. Therefore, the aim of our research study was to assess the potential color-masking ability of all four tooth colored restorative materials—GIC, RMGIC, composite, and Cention N—over stained 38% SDF using a UV‒VIS-NIR spectrophotometer. The null hypothesis states that there will be no color masking with four different tooth-colored restorative materials over stained 38% SDF.

## Materials and methods

This in vitro laboratory study was initiated after approval from the Institutional Ethics Committee, Manipal College of Dental Sciences, Mangalore, under the number IEC- 22061. The International guidelines, as mentioned in the Declaration of Helsinki, were followed by the authors prior to the usage of human body material in medical research.

### Study setting

This study was conducted in the Department of Pediatric and Preventive Dentistry at Manipal College of Dental Sciences, Mangalore, and the Central Research Facility, National Institute of Technology Karnataka, Surathkal.

According to the study by Hamdey et al. [8], the standard deviation expected is 8.35, with a 5% of alpha error and a power of 95%, and maintaining an effective difference to show a clinically significant difference of 20 units, a sample size of 5 extracted primary teeth was used for each group. The formula used for sample size calculation is-

$$N=\frac{{2\left({Z}_{1-\frac{\alpha }{2}}+{Z}_{1-\beta }\right)}^{2}{\sigma }^{2}}{{d}^{2}}$$

Therefore, a total of 20 teeth were collected those indicated for extraction and placed in distilled water. The study samples were randomly divided into four groups.

**Table:** 

GROUP A (*n* = 5)	38% SDF (e SDF) + GIC (GC gold label)
GROUP B (*n* = 5)	38% SDF (e SDF) + RMGIC (GC Fuji plus)
GROUP C (*n* = 5)	38% SDF (e SDF) + COMPOSITE (3 M)
GROUP D (*n* = 5)	38% SDF (e SDF) + CENTION N (Ivoclar)

Extracted deciduous carious teeth with occlusal caries extending to the dentin with ICDAS scores of 5 and 6 that had been recommended for extraction for periodontal, orthodontic, or caries-related causes were included in the study, and deciduous carious teeth with ICDAS scores other than 5 and 6 were excluded.

### Tooth preparation

Teeth were cleaned thoroughly to remove any plaque and debris using a low-speed handpiece and then mounted in dental stone. Thereafter, distilled water was used for storing the teeth at room temperature.

First, 38% SDF was applied to all the teeth according to the manufacturer’s instructions. One drop of SDF was applied for 1 min directly over the carious dentin using a microbrush, after which the teeth were washed and dried [8]. After some time, black discoloration was observed, after which a baseline reading was taken before adding any test restorative materials.

### Placement of tooth-colored restorative material

The 20 tooth specimens were divided randomly into 4 groups for restoration.

**Fig. 1:**
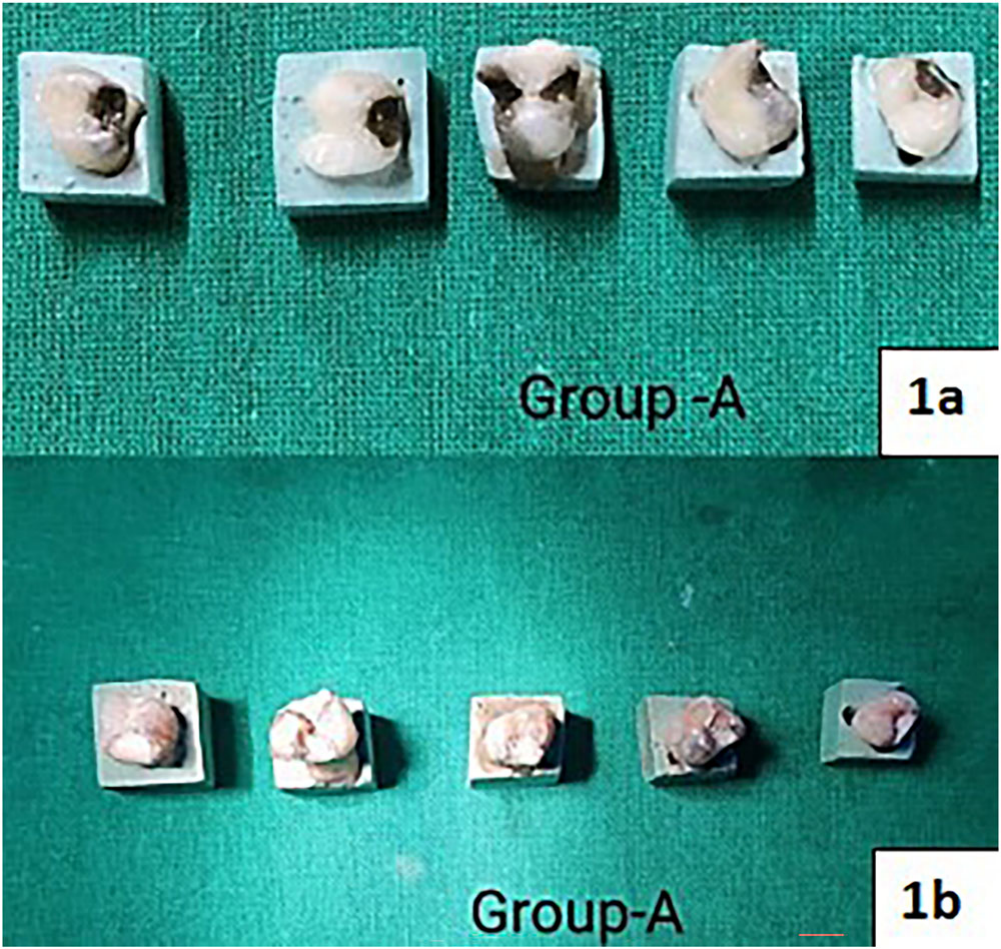
Group A:(**1a**) Application of 38% SDF; (1**b**) Restoration with conventional GIC.

**Fig. 2:**
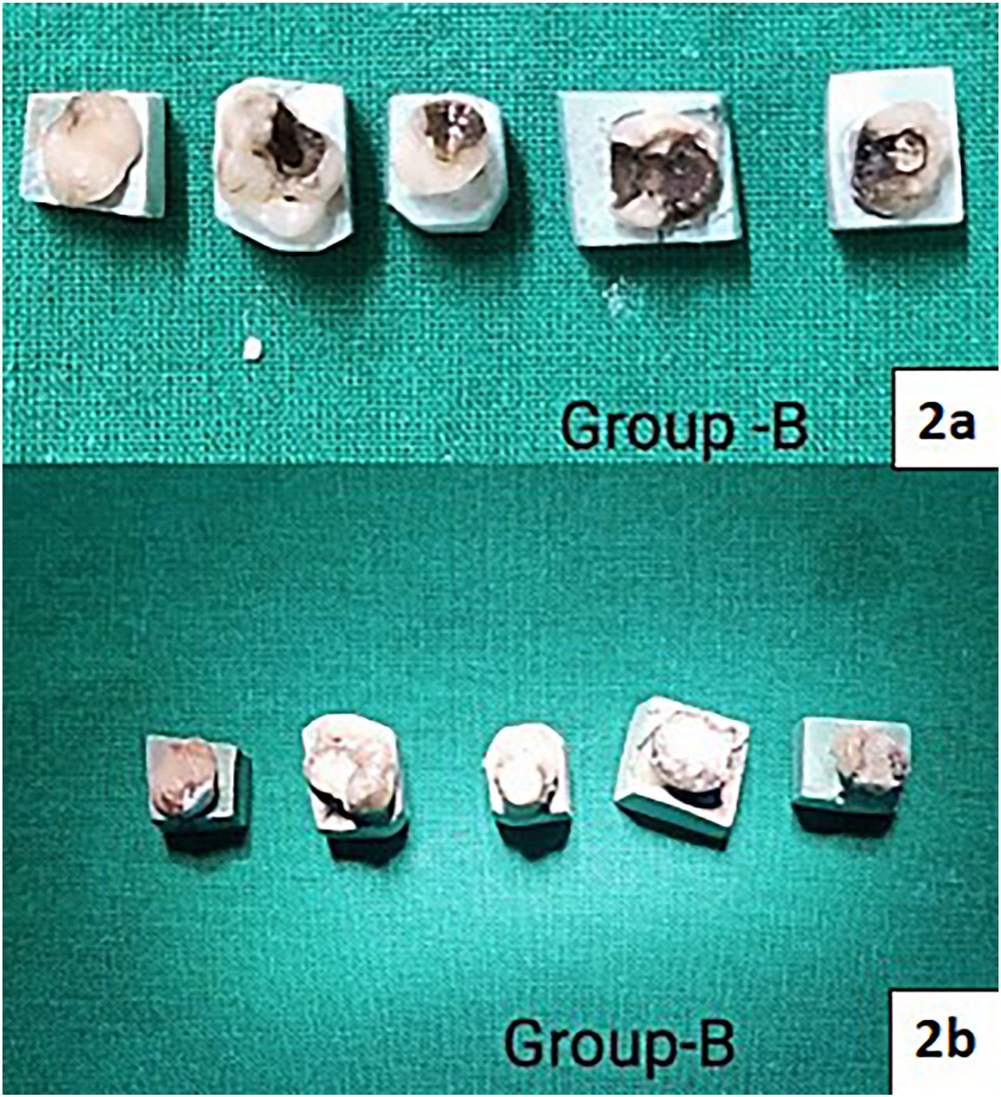
Group B:(**2a**) Application of 38% SDF; (**2b**) Restoration with RMGIC.

**Fig. 3:**
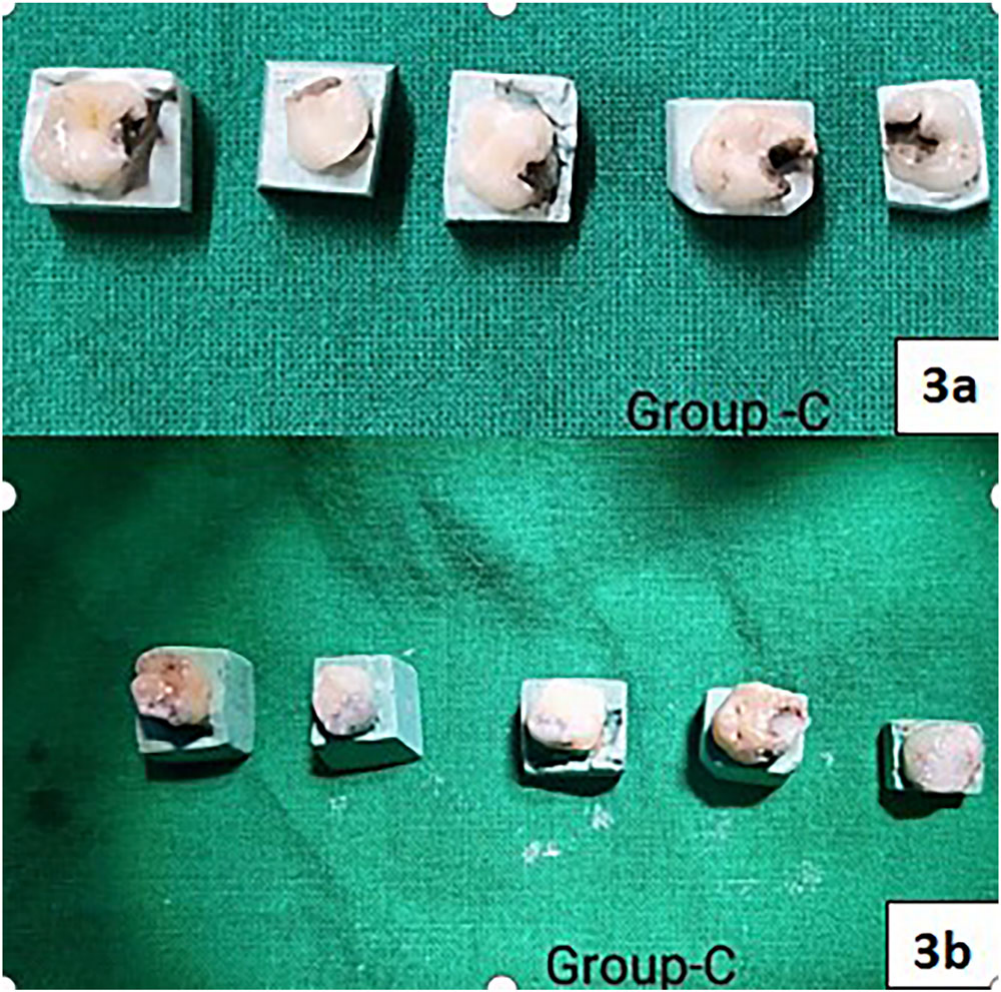
Group C:(**3a**) Application of 38% SDF; (**3b**) Restoration with Composite.

**Fig. 4:**
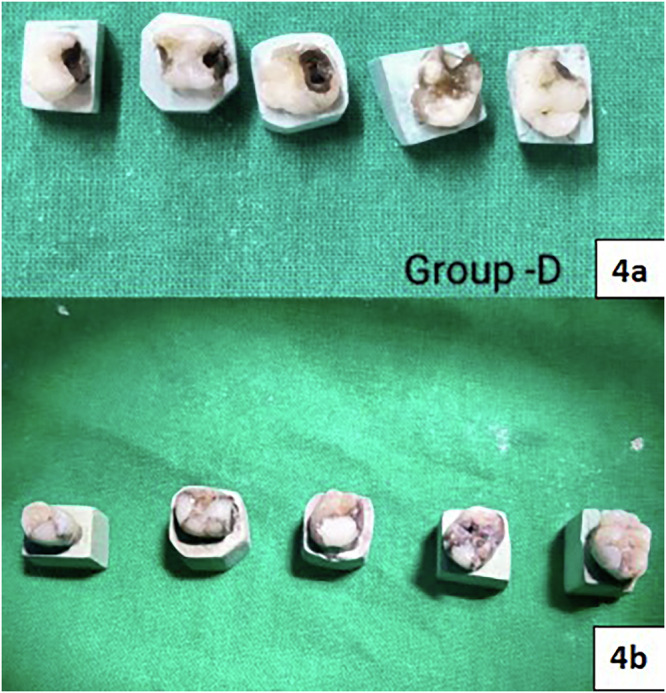
Group D:(**4a**) Application of 38% SDF; (**4b**) Restoration with Cention N.

For Group A, the teeth were conditioned for 20 seconds, after which the GIC was restored. (Fig. 1) In Group B, the RMGIC was placed and for 20 seconds light cured [9] (Fig. 2). In Group C, 37% phosphoric acid was applied for 20 seconds for etching and then washed and dried followed by application of bonding agent, and then light cured [10] (Fig. 3). In Group D, Cention N restorative material was mixed and added. All the restorations were 2 mm thick, and the shade of the restorative material used for all the above groups was A1 [11] (Fig. 4).

The color of all the test materials was recorded immediately after placement.

### Color measurement using a spectrophotometer

Color was measured using a UV‒VIS-NIR spectrophotometer with reflectance spectra (200 nm-1000 nm). The coated sample was directly measured following a previous calibration. The device was adjusted and calibrated daily on black and white calibration tiles. The expected peaks of silver (Ag) were investigated between 350 and 450 nm, while the enamel peaks recorded in the former spectra were ignored. Two readings were recorded for each tooth specimen: baseline=1 and directly after restoration placement=2.

ΔL, Δa, and Δb were calculated by measuring the L*, a*, and b* values (L*= lightness, + a* = red, − a* = green, + b* = yellow, − b* = blue). Then, the following equation was used to assess the extent of the color change (ΔE) [12].

$${{\Delta }}{{{\rm{E}}}}=\surd {({{{\rm{L}}}}1-{{{\rm{L}}}}2)}^{2}+{({{{\rm{a}}}}1-{{{\rm{a}}}}2)}^{2}+{({{{\rm{b}}}}1-{{{\rm{b}}}}2)}^{2}$$

Statistical analysis was conducted with SPSS 24. To check the color-masking ability of each restorative material, multivariate analysis of variance (MANOVA) was applied. In this study, T2-T1 were evaluated. Intergroup comparisons were performed using the Schefe post hoc test.

## Results

The intragroup comparison for color masking ability (ΔE) for all four restorative materials revealed no statistically significant differences, with a test value of 1.168 and a p value of 0.353, and the highest mean was observed in Group A, i.e., 38% SDF + GIC (9.171966), followed by Group D, i.e., 38% SDF+ Cention N (7.977943); Group C, i.e., 38% SDF+ Composite (7.865261); and Group B, i.e., 38% SDF + RMGIC (2.356184). i.e., GIC, RMGIC, composite, and Cention N. All these materials were equally effective at masking the black discolouration caused by SDF application. Therefor, thee null hypothesis was accepted.

Regarding the red/green scale (Δa), a significant difference in the color shift was observed between all four groups, with more shifting toward green color observed for Group C (−0.712 ± 0.54), followed by Group B (−0.104 ± 0.519); however, Group A and Group D were red in color (Table 1).

**Table 1:** Intergroup comparison of the color-masking ability of different restorative materials.

	Group A (38%SDF + GIC)	Group B (38%SDF + RMGIC)	Group C (38% SDF+ Composite)	Group D (38%SDF+ Cention N)	F/welch	*P* value
**L1**	85.176 ± 1.127	84.438 ± 1.504	84.31 ± 10.283	78.338 ± 7.052	1.443*	0.301
**a1**	0.1 ± 0.183	0.122 ± 0.26	0.254 ± 0.442	0.304 ± 0.116	1.559*	0.271
**b1**	0.236 ± 0.827	0.708 ± 0.643	0.592 ± 0.318	0.322 ± 0.747	0.562	0.648
**L2**	76.664 ± 8.213	83.04 ± 2.335	86.396 ± 3.741	84.314 ± 1.592	3.934	0.028
**a2**	0.592 ± 0.701	−0.104 ± 0.519	−0.712 ± 0.545	0.088 ± 0.192	5.305	0.01
**b2**	2.488 ± 2.957	−0.088 ± 0.34	−0.062 ± 0.5	1.686 ± 0.559	12.246*	0.002
**Δ E**	9.172 ± 9.177	2.356 ± 1.579	7.865 ± 6.524	7.978 ± 5.468	1.168	0.353

Regarding lightness (L), red/green scale (Δa) and yellow/blue scale (Δb), significant differences in color were observed, with more shifts toward yellow in group A (2.488 ± 2.957) and group D (1.686 ± 0.559) and more shifts toward green in groups B (-0.088 ± 0.34) and C (−0.062 ± 0.5). The mean lightness was greatest for Group C, i.e., the composite group (86.396 ± 3.741), and least for Group A, i.e., the GIC group (76.664 ± 8.213), indicating lighter tooth shade with the composite on SDF tooth discoloration compared to the other restorative materials (Table 1).

**Fig. 5:**
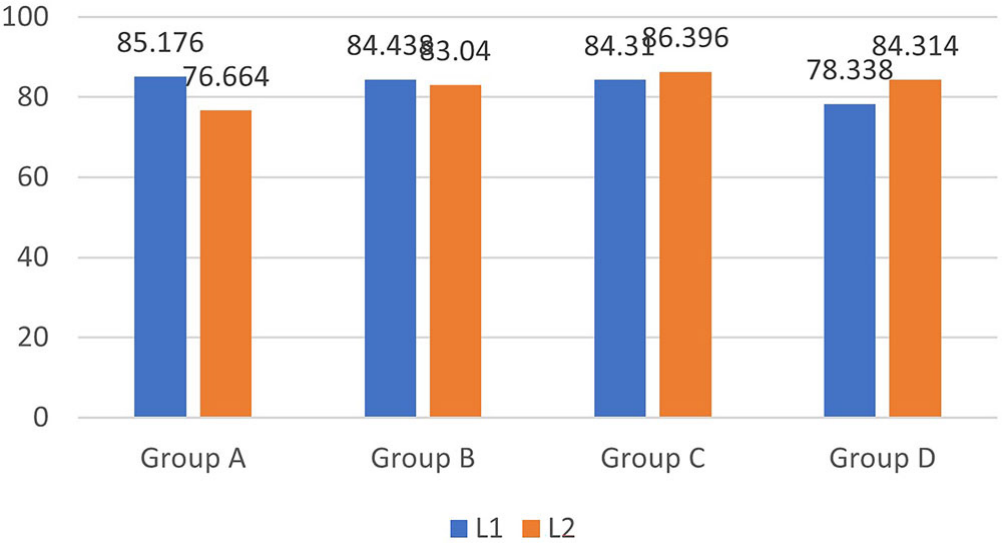
Comparison of Lightness among the Four Restorative Materials.

**Fig. 6:**
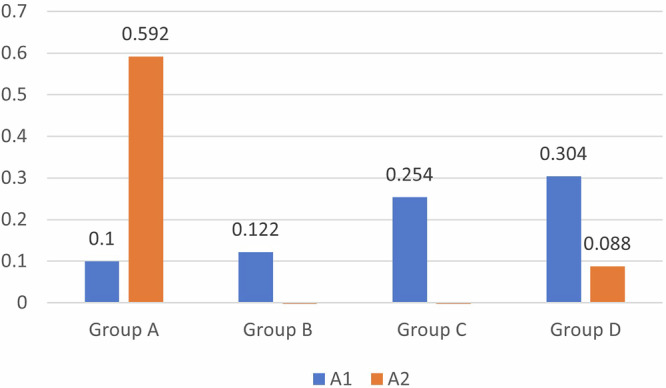
Comparison of the red/green scale (a*) between the four restorative materials.

**Fig. 7:**
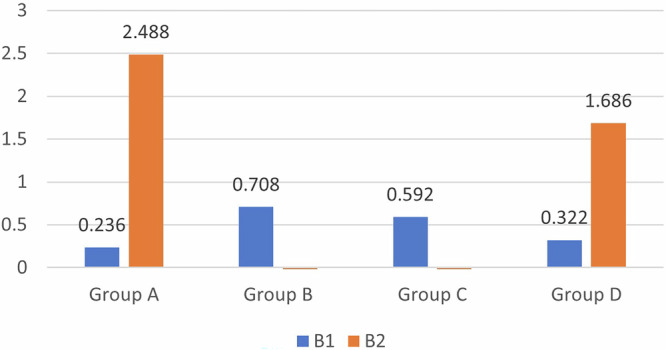
Comparison of yellow/blue scale (b*) between the four restorative materials.

A comparison of the mean values for Group A- 38% SDF + GIC, L1 and L2 revealed that the mean value of L1 was greater, with a difference of 8.512, which was not statistically significant, with a *p*-value of 0.1. (Fig. 5) A comparison of the mean values of A1 and A2 revealed that the mean value of A2 was greater, with a difference of 0.492; this difference was not statistically significant, with a *p*-value of 0.189. (Fig. 6) A comparison of the mean values of B1 and B2 revealed that the mean value of B2 was greater, with a difference of 2.252; this difference was not statistically significant, with a *p*-value of 0.218.(Fig. 7) For Group B- 38% SDF + RMGIC, a comparison of the mean values of B1 and B2 shows that the mean value of B1 is greater, with a difference of 0.796, which is statistically significant, with a *p*-value of 0.029. In Group C- 38% SDF+ composite, the mean values of A1 and A2 revealed that the mean value of A1 was greater, with a difference of 0.966, which was statistically significant, with a *p*-value of 0.006. A comparison of the mean values of B1 and B2 shows that the mean value of B1 is greater, with a difference of 0.654, which is statistically significant, with a *p*-value of 0.031. The results for Group D- 38% SDF+ Cention N showed that in comparison with the mean values of B1 and B2 shows that the mean value of B2 is greater, with a difference of 1.364, which is statistically significant, with a *p*-value of 0.022 (Table 2).

**Table 2:** Intragroup comparison of the color-masking ability of different restorative materials

			*N*	Mean ± SD	Mean difference ± SD	T	*P* value
Group A **(38%SDF** + **GIC)**	Pair 1	L1	5	85.18 ± 1.13	8.51 ± 8.93	2.13	0.1
		L2	5	76.66 ± 8.21			
	Pair 2	a1	5	0.1 ± 0.18	−0.49 ± 0.69	−1.58	0.189
		a2	5	0.59 ± 0.7			
	Pair 3	b1	5	0.24 ± 0.83	−2.25 ± 3.45	−1.46	0.218
		b2	5	2.49 ± 2.96			
Group B (**38%SDF** + **RMGIC)**	Pair 1	L1	5	84.44 ± 1.5	1.4 ± 2.39	1.31	0.262
		L2	5	83.04 ± 2.34			
	Pair 2	a1	5	0.12 ± 0.26	0.23 ± 0.34	1.48	0.214
		a2	5	−0.1 ± 0.52			
	Pair 3	b1	5	0.71 ± 0.64	0.8 ± 0.53	3.33	0.029
		b2	5	−0.09 ± 0.34			
Group C **(38% SDF+ Composite)**	Pair 1	L1	5	84.31 ± 10.28	−2.09 ± 10.6	−0.44	0.683
		L2	5	86.4 ± 3.74			
	Pair 2	a1	5	0.25 ± 0.44	0.97 ± 0.41	5.24	0.006
		a2	5	−0.71 ± 0.55			
	Pair 3	b1	5	0.59 ± 0.32	0.65 ± 0.45	3.27	0.031
		b2	5	−0.06 ± 0.5			
GroupD **(38%SDF+ Cention N)**	Pair 1	L1	5	78.34 ± 7.05	−5.98 ± 7.85	-1.70	0.164
		L2	5	84.31 ± 1.59			
	Pair 2	a1	5	0.3 ± 0.12	0.22 ± 0.29	1.68	0.167
		a2	5	0.09 ± 0.19			
	Pair 3	b1	5	0.32 ± 0.75	−1.36 ± 0.83	−3.66	0.022
		b2	5	1.69 ± 0.56			

The mean difference in group B, i.e., the RMGIC group, was significant on the yellow/blue scale (b*) with a shift toward yellow. This indicates that RMGIC provides a lighter shade when applied for SDF discoloration. Group C, i.e., the composite group, showed significant results on both the red/green (a*) and yellow/blue (b*) scales, with a shift seen toward the red and yellow scales indicating that it was redder and yellower and lighter, respectively, when placed on SDF discoloration. Group D, i.e., the Cention N group, again showed significant results on the yellow/blue scale (b*); however, a shift was observed toward the blue scale.

## Discussions

SDF is an alkaline solution, colorless, and contains fluoride and silver and forms a mixed heavy-metal halide coordination complex when combined with ammonia. Silver and fluoride together have the ability to simultaneously stop the progression of caries and prevent the formation of new caries by decreasing the colony forming unit (CFU) counts of Actinomyces naeslundii and *S. mutans* mono-species strains. Moreover, SDF increases the mineral content of hard tooth tissues and facilitates calcium absorption. Therefore, it is known that carious lesions treated with SDF have a much greater surface microhardness [4]. The application of SDF is considered as an alternative and effective way to manage dental caries. Two concentrations of SDF are available on the market: 12% and 38%. A study conducted by Fung et al. [13], showed that, compared with 12% SDF, 38% SDF led to an 18–20% increase in the rate of caries arrest and had fewer adverse effects. Therefore, the same concentration of SDF, i.e., 38%, was used in our study. In a study conducted by Vennela E et al. [6], ICDAS score 5 and 6 extracted teeth were included for application of SDF and also in an in vivo study by H. M. Abdellatif et al. [14], SDF was compared with atraumatic restorative treatment (ART) on primary teeth with ICDAS score 4, 5, 6 and concluded that both SDF and ART are, indeed, effective for arresting caries. Nonetheless, a study by Rossi et al. [15], investigated the impact of SDF on the pulp complex by looking at experimental trials on lab animals as well as a histological analysis of human teeth treated with SDF. Microscopy revealed that SDF only sealed dentinal tubules at the location where it was applied, with little penetration underneath, in the ex vivo examination of human teeth. There was no Ag precipitate in the pulp tissue connected to treated caries, but there was a persistent inflammatory infiltration and tertiary dentine development. According to the study’s findings, SDF has very little negative impact on pulp. Therefore, ICDAS score of 5 and 6 [16] were included in our study.

The use of any restorative material depends critically on color acceptability [17]. The major disadvantage of SDF is tooth discoloration; therefore, how to lessen this discoloration is unknown. A number of studies have attempted to gauge parents’ acceptance of their children using SDF [18]. Due to discoloration issues, only 53.3% of parents thought that SDF treatment is appropriate for their child [19]. Parental approval of SDF therapy was shown to be much greater for posterior teeth than for anterior teeth, according to research by Sabbagh et al. [18].

Studies have shown that the long-term success of SDF depends on its restoration. Various restorative materials can be used over SDF. In our study, we used 4 restorative materials—self-cured GIC, RMGIC, composite, and Cention N which are the most commonly used restorative materials in children.

In a study conducted by Raafat et al. [20], the highest ΔE value for RMGIC indicated that it was least effective in color masking, and the lowest value for zirconia-reinforced glass ionomer cement indicated that it was better in color masking. This result was in contrast to our study, in which our study showed similar effects for all four restorative materials; however, RMGIC had the lowest ΔE value, indicating that it was better than rest, and the highest ΔE value was noted for self-cured GIC. This difference could be due to the consideration of different cavities in both studies. In our study, we included class I and II cavities, and in a study by Raafat et al. [20], class V cavities were included, and different tooth-colored restorative materials were considered.

Vivek Sharma et al. [21], stated that value is considered more important than other components, such as hue and chroma. Our study revealed that the RMGIC group was more yellower and that the Composite group was more lighter/redder/yellower, indicating that these 2 restorative materials are lighter in the shade when used on SDF discolored teeth and that the Cention N group was bluer, indicating that it provided a darker shade over SDF discolored teeth [22].

The result of our study showed that RMGIC group, was significant on the yellow/blue scale (b*) with a shift toward yellow. This indicates that RMGIC provides a lighter shade when applied for SDF discoloration. Group C, i.e., the composite group, showed significant results on both the red/green (a*) and yellow/blue (b*) scales, with a shift seen toward the red and yellow scales indicating that it was redder and yellower and lighter, respectively, when placed on SDF discoloration. Group D, i.e., the Cention N group, again showed significant results on the yellow/blue scale (b*); however, a shift was observed toward the blue scale. This indicates that Cention N is a bluer and darker shade for SDF discoloration [23]. Additionally, in people with fair skin, a lower degree of tooth shade was found, resulting in teeth that looked darker in color. Viceversa with darker skin and greater tooth shade were found, leading to teeth that seemed lighter in color. Additionally, when comparing the tooth colors of men and women, a lower shade value was observed for men/boys, and a greater shade value was observed for women/girls [24]. Future in- vivo studies can be carried out to assess the type of restorative material suitable based on gender and skin tone on SDF discolored tooth.

A few limitations of this study could be that it is an in vitro study, which limits the extrapolation of the results to the oral ecosystem [25]. Additionally, distilled water was used as storage media in our study, and dry specimens were used during the experimental phase; therefore, the degree of staining may be different in the oral environment because of the presence of saliva compared to our study and since SDF discoloration intensifies over time [26] future studies can be conducted to assess color masking of different restorative materials at different time intervals.

## Conclusions

Within the limitation of the study, it is concluded that all 4 restorative materials were equally effective in terms of color masking, with 38% SDF discoloration.

## Data Availability

The data generated and analyzed during the present study are not publicly available due to ethical restrictions but are available from the corresponding author upon reasonable request.
